# Communication and the Emergence of Collective Behavior in Living Organisms: A Quantum Approach

**DOI:** 10.1155/2013/987549

**Published:** 2013-10-30

**Authors:** Marco Bischof, Emilio Del Giudice

**Affiliations:** ^1^Institute for Transcultural Health Sciences, European University Viadrina, Grosse Scharrn Strasse 59, 15230 Frankfurt (Oder), Germany; ^2^Retired Scientist from Istituto Nazionale Fisica Nucleare (INFN), via Celoria 16, 20133 Milano, Italy

## Abstract

Intermolecular interactions within living organisms have been found to occur not as individual independent events but as a part of a collective array of interconnected events. The problem of the emergence of this collective dynamics and of the correlated biocommunication therefore arises. In the present paper we review the proposals given within the paradigm of modern molecular biology and those given by some holistic approaches to biology. In recent times, the collective behavior of ensembles of microscopic units (atoms/molecules) has been addressed in the conceptual framework of Quantum Field Theory. The possibility of producing physical states where all the components of the ensemble move in unison has been recognized. In such cases, electromagnetic fields trapped within the ensemble appear. In the present paper we present a scheme based on Quantum Field Theory where molecules are able to move in phase-correlated unison among them and with a self-produced electromagnetic field. Experimental corroboration of this scheme is presented. Some consequences for future biological developments are discussed.

## 1. Introduction

A living organism is fundamentally different from a nonliving system. There are basically two main differences. The first one is the capability of self-movement; namely, a living organism is able to pursue autonomously the direction of its own motion, whereas a nonliving object can be only pushed or pulled by an externally applied force. The second difference is that the dynamics of each component depends on the simultaneous dynamics of the other components, so that the ensemble of components behaves in unison in a correlated way. It is just this collective dynamics which makes possible the self-movement of the system, allowing a continuous change of the organism without disrupting its fundamental unity. This property is missing in nonliving systems which are fundamentally passive. The main actor of the time evolution of the organism is not the ensemble of molecules but the ensemble of their correlations. 

For this reason, we cannot assume that the molecular components of a living organism are independent molecules moving in a diffusive way, but we are forced to assume a holistic dynamics able to preserve the unity of the organism amidst a huge number of externally applied stimuli and challenges. In particular, molecules encounter each other in the organism not at random but apparently according to “organic codes” evolving in space and time, as, for example, the genetic code [1]. This means that molecules which, taken individually, can interact chemically with any kind of other molecules, acquire within the living organism the property of selecting their chemical partners according to codes evolving in time.

In this framework, the problem of communication becomes a crucial issue in the understanding of living organisms. The existence of this array of molecular communications, able to adapt to a wide number of external stimuli and constraints, is a permanent feature of every organism which disappears at death only. The array of communications among molecules does not depend on external causes but is inherent in the modus operandi of the molecular dynamics. In other words, the spreading of information about each event depends on the same dynamics which produces the event. Biochemical reactions and the spreading of information about them should be two different aspects of the same dynamics and in particular do not need additional expenses of energy. 

The problem of biocommunication has been addressed in recent times within the frame of the molecular paradigm, which states that a living organism is an ensemble of appropriate molecules kept together solely by chemical forces, whose essential features are that they can be always reduced to pairwise interactions. In this frame, the problem of communication has been reduced to the ligand-receptor model as described in Section 2. In Section 2 we will describe the historical development of the attempts to this kind of explanation. However, the final result is unsatisfactory at least for two reasons. First, the existence of chemical codes remains unexplained since no reason is given why a molecule is able to encounter its molecular partner in the sequence underlying the given biological cycle just in the right place at the right time; this would demand a long-range recognition and attraction among molecules. The second reason is that the spreading of information about each molecular event to the other component molecules of the organism would require the emission of signals, such as chemical messengers or electromagnetic signals, whose formation would require energy. The huge ensemble of all the signals necessary to keep other parts of the organism informed about what is going on in one part, so that they are able to act in an holistic way, would demand an immense consumption of energy. On the contrary, we know that the energy demands of an organism are quite moderate [2]. 

Therefore we are compelled to opt for a different paradigm, which is offered naturally by modern quantum field theory [3–5]. According to Quantum Physics, each object cannot but fluctuate; not only material particles but also fields are undergoing spontaneous fluctuations, that is, fluctuations occurring without any external supply of energy. Under conditions clarified by the theory which we will shortly describe in Section 4 the quantum fluctuations of many objects are able to correlate with their phases, producing a common oscillation of all the components in unison. The system therefore enters into a state which in the physical jargon is termed “coherent state”. The energy of an ensemble of components in a coherent state is lower than the energy of the same ensemble in a noncoherent state, since the onset of coherence eliminates all the “useless” movements of components which give rise to the entropy of the system and concentrates the energy on a smaller number of degrees of freedom able to use this energy to produce external work [6–9]. This property of coherent systems could implement the requirements of Prigogine for dissipative structures [10]. In order to abide by the second law of thermodynamics, the process of energy concentration demands that some energy should be released outwards, so that, in order to become coherent, a system should be an open system. In a coherent system made up of atoms/molecules, there is therefore a physical field able to keep the long-range correlation among the components [11]. This correlation field can be shown to be just the electromagnetic potential, whose space-time derivatives give rise to the well-known electromagnetic fields [12]. Therefore, what keeps the molecular components correlated among them not in a pairwise way but in a truly collective many-body way is not the electromagnetic field whose production would demand energy but the electromagnetic potential whose appearance in a coherent system produces a net saving of energy [13–17]. The physical variable responsible for correlation in this vision is not the energy but the phase of the system. We will discuss this point further in Section 3. Recently, strong experimental evidence about the existence of coherent correlations among biomolecules and the role of electromagnetism in biocommunication has been reported [18, 19].

The perspective outlined above emerges from modern Quantum Physics whose validity in the understanding of elementary particles, atoms, and molecules has been fully established in the last century. Moreover, this approach, contrary to the physics of the past, either Classical Physics or old quantum mechanics, does not allow us to address physical objects in isolation but only as parts of an extended reality, the field, where its connectedness with other objects is pivotal. The communications among all the components of the physical systems occur via quantum fluctuations which cannot be reduced to the actual fluctuations of the components but also include the fluctuations of the vacuum. The vacuum therefore could be assumed to be an agent for the spreading of communication among components [20]. Quantum Field Theory only recently has been able to get out of the microscopic world of elementary particles and has been used to analyze macroscopic systems too, such as crystals and superconductors. It can be proposed as a good candidate for understanding living organisms [3–5]. 

The aim of the present paper is to provide first a concise overview of the historical development of the problem of biocommunication and of the formation of a collective dynamics in living organisms, second to point out the deficiencies of the purely molecular approach and the need for coherence in the living dynamics, and third to sketch the conceptual framework offered by Quantum Field Theory for the understanding of the onset of coherence in matter and living organisms. 

The paper is organized as follows. In Section 2, we will describe the problem of biocommunication and the emergence of collective behavior within the molecular paradigm of biology. In Section 3, we will discuss the contributions offered by approaches to biology different from molecular biology; in Section 4 we will introduce and discuss the possibilities provided by the quantum field theoretical approach, in Section 5 we present some experimental corroborations for this approach, and finally in Section 6 we will draw some conclusions and provide some outlook. 

## 2. The Molecular Approach to Biocommunication

Biological communication conventionally is assumed to be entirely chemically mediated. Modern views still are fundamentally based on Ehrlich's ligand-receptor model stating that biological reactions can only be elicited by messenger molecules (such as hormones and neurotransmitters) binding to receptors (which can be cell-membrane bound ones, cytoplasmic proteins, or other molecules). Clark's “receptor occupation theory” in 1933 postulated four rules for the action of transmitter substances on receptors: (1) the effect increases proportionally with the number of occupied receptors; (2) for each receptor's action an “all-or-nothing” response applies; (3) substrate molecule and receptor fit in a “lock-and-key” fashion; and (4) the occupation of one receptor does not interfere with the function of other receptors [21].

Although a great number of bioreceptors have been found, for a number of reasons there has been growing criticism of this model [22–25]. This static and mechanistic approach implies biological response to be strictly local and linear (the response is proportional to the stimulus). Already the first attempt to extend the model, undertaken in 1961 by Paton, implicitly questioned the receptor hypothesis. Paton's “rate theory,” which is in much better agreement with experience, states that the number of contacts between receptor and transmitter molecules is essential for biological efficacy [26]. Thus chemical communication acquires a fundamental dynamic aspect. Secondly, the control of the dissociation and association rate of the receptor-transmitter complex becomes a central part of the regulatory function. Thus, communication is not any more restricted to the structural level, that is, the structural manifold of biochemical messenger molecules; any factor able to influence the coupling of molecules (i.e., an electromagnetic coupling) has to be taken into consideration. 

The regulation of the binding activity as well as that of subsequent steps that typically involve signal amplification toward some biological response is today thought to be chemical as well. But the discovery of chemical messengers and receptors, which doubtlessly has greatly enlarged biological understanding, may not be the last word about biological communication. They may turn out to be necessary but not sufficient mediators of cellular communication. Their identification still leaves open some essential questions of biological communication, such as: What coordinates the biological processes? What directs the right number of molecules needed at the right time to the right place? How does the coupling to the receptor trigger the ensuing response? Also, there are indications that the speed of the signal conveyed at least in some biological communication processes clearly exceeds the speed range of molecular transmission. 

The biochemical model of signal transmission clearly needs a biophysical complement. Such a complement has been proposed for the surface receptor-cytoplasm communication across the membrane. According to Adey, the glycoproteins on the cell surface which mediate the cell-cell recognition also can act as antennae for e.m. signals [27]. After the transmission across the membrane, the signal is propagated to the cytoplasm by cytoskeleton-mediated events, in which again e.m. processes may be involved [28, 29].

The same doubts apply to the coupling of molecules in biochemical reactions. The task of studying the individual biochemical reactions *in vitro* has been so enormous and absorbing that little work has been devoted to the question of how the reactions are organized and how the reactants are moved *in vivo*. As Rowlands remarks, it is inconceivable that the cell has to wait for the chaotic Brownian motion (and diffusion, for this matter) to accomplish these things by chance [30]. Therefore we must come to the same conclusion as Szent-Gyorgyi [31] who stated that biologists might not be able to formally distinguish between “animate” and “inanimate” things because they concentrate on studying substances to the neglect of two matrices without which these substances cannot perform any functions—water and electromagnetic fields.

### 2.1. Intermolecular and Intercellular Recognition

Already Weiss has pointed to the fact that cells can recognize each other and their surroundings and can find their proper destinations in the organism even when customary routes are blocked [32, 33]. Additionally, cells of the same type tend to aggregate and actively preserve this aggregation not only in the body but also  *in vitro*. Mutual recognition between cells of the same type later has been shown for dissociated liver and kidney cells from a vertebrate embryo [34]. The aggregation of identical cells (liver-liver, kidney-kidney) takes place at a faster rate than that of the mixed cells. The mechanisms of this tissue-specific recognition are not yet understood. Rowlands found that erythrocytes actively attract each other to form rouleaux when the cell membranes are still 4 *μ*m apart, a distance ten thousand times the range of the known chemical forces [35, 36]. He suggests that long-range cell interactions of this type are also responsible for the very fast attraction of platelets to the cells of a damaged cell wall and may also underlie the ability of leukocytes to actively look for and destroy elements foreign to the host organism. Bistolfi proposes that this effect may explain the preference of neoplastic metastases for certain target organs [25]. Weiss in 1959 has asked the questions that biology today still has not been able to answer satisfactorily [32, 33]: How do mixed cell populations achieve this? Do like cells attract each other? Do they recognize each other only after chance encounters? Weiss was convinced that these phenomena are of the same general nature than immune reactions, enzyme-substrate interactions, the pairing of chromosomes, fertilization, parasite infection, and phagocytosis. 

It may well be that we are dealing with a mechanism that is even more general and, on the microscopic side, also extends to atomic and molecular interactions and to interactions between organisms on the macroscopic side. Already Presman has proposed that a long-range e.m. mechanism may be responsible for the recognition between macromolecules, such as between enzyme and substrate, DNA and RNA, and antigen and antibody, and between cells [37]. According to Rowlands the phenomenon of erythrocyte attraction shows the existence of a species-specific, electromagnetic ultralong-range interaction, a mechanism by which similar cells may recognize each other and join up [36]. Paul has explained these long-range forces between human blood cells by the use of coherent states of the e.m. field [38]. An interpretation of these phenomena in terms of a coherent dynamics has been proposed by Del Giudice et al. [29, 35]. 

As we will see, such a mechanism of e.m. communication on all levels of biological organization constitutes an essential element of an electromagnetic theory of the living organism. Biological functions such as the control of enzymatic activity, photorepair, and immunological functions, particularly, are obviously based on some process of pattern recognition and thus suggest understanding in terms of coherent interactions [39].

### 2.2. Long-Range e.m. Forces in Molecular Interaction

As to the microscopic level, we need a new way to look at molecular interactions, which fits to the field picture of life. Of the many factors able to influence the coupling of molecules, the electromagnetic field may be the essential one. As Bistolfi pointed out, the chemical structure of biomolecules is not in itself enough to characterize intermolecular recognition interactions [25]. The wide range of chemical shift values as determined by NMR shows that organic molecules not only differ in their chemical structure but also in the way they resonate when adequately stimulated, that is, in their electromagnetic patterns. Thus Bistolfi postulates that resonance capacity in a more general sense than that appearing in NMR spectroscopy must be considered—since not only the paramagnetic nuclei but also the molecular structures are involved in it. Bistolfi takes this evidence from NMR to be a good indirect indication of the existence of long-range, frequency-dependent e.m. intermolecular interactions. Frazer and Frazer postulate that e.m. radiation emitted by functional groups on molecules induces specific orientations/conformations/alignments in complementary molecules and thereby generates an electromagnetically induced geometrical “template” of the two molecular regions [40].

Molecules themselves have to be considered as ordered electromagnetic structures [41, 42]. Their shape and stability depend on the delicate balance of e.m. interactions between neighbouring atoms. The main factors in molecular interaction, in the conventional quantum chemical view, are the spatial conformation of the molecules involved and the charge distribution of the valence electrons (chemical potentials). It is primarily the charge distribution on the surface of molecules that determines their physical and chemical properties, such as bonding ability, orientation, and mutual position. When the geometrical configuration of two molecules fits in such a way that a minimum of the (electrical) Coulomb potentials of the valence electrons is achieved, a chemical bonding can take place. However, charge distribution through its time variations is also responsible for the properties of the e.m. radiation which molecules emit, such as polarization, spatial distribution, and direction, and for their interaction with impinging radiation. The interaction of radiation with matter is only possible through redistribution of charge. Structural changes in the molecules entail charge shifts and thus changes in the e.m. field envelope of the molecule, which may be the real mediator of the molecule's interaction with other molecules. 

A field approach to intermolecular interaction is fundamental to any electromagnetic model of living organisms. Li suggests using the De Broglie wave picture of matter in looking at nonradiative interaction between particles [43–45]. In this view, atomic orbits represent interference patterns of electron waves within their coherence (or uncertainty) volume. The attractive superposition of the quantum-mechanical wave functions, in the case of the bonding of two hydrogen atoms, is identified to destructive interference of their electron waves, while the repulsive superposition of the wave functions, in the antibonding case, is equated to the constructive interference of the electron waves. In atoms and molecules thus the same mechanism is seen at work as that used to explain Galle's observations on the ultraweak bioluminescence of Daphnia aggregations [46]. According to Popp's biophoton theory, the same kind of approach can be used for the case of atoms and molecules influencing each other only by means of the radiation field, when, for instance, the same two atoms are sufficiently separated that no electron wave function overlap is possible [43–45]. As long as two (or more) radiating atoms or molecules remain within the coherence length, they can be arbitrarily far apart and still exhibit some correlation effect. Within the coherence volume, which is the region within which the wave remains coherent, the photons emitted are completely delocalized. The exchange of photons between the particles builds interference patterns, the basis of a communication linkage among the particles, which thereby form a complex cooperative system that has to be considered as a whole. As well as to the interaction between atoms and molecules, this model applies to the interaction between macromolecules and cell components, between cells, and between organisms in populations. 

 More recently, Irena Cosic has presented a “Resonant Recognition Model” of biomolecular interaction which postulates that these interactions are of electromagnetic nature [41, 42, 47, 48]. Based on the finding that proteins produce electromagnetic emission and absorption in the range of infrared and visible light, Cosic proposes that molecules recognize their targets by electromagnetic resonance and that electromagnetic waves are also used by them to influence each other at a distance and to attract molecular partners. By means of electromagnetic resonance, selective interactions are taking place at distances greatly exceeding the range of chemical interactions. However, even if it brings a laudable progress in the right direction, Cosic's model is fraught with the serious deficiency of not considering the actual physical value of the photon-atom cross-section governing the electromagnetic interaction of atoms, which is in fact quite small. If the e.m. field is assumed to be, as in the usual case, a flow of photons travelling at the speed of light and therefore having a small probability of encountering atoms, the Cosic proposal has a small chance of explaining the actual biological interaction. However, the Cosic proposal assumes a very important value when it is included within a dynamics able to put, in a sense, the e.m. field “at rest,” that is, to trap it within a self-produced cavity, as we will see in Section 3. In this case, the coupling between the e.m. field and the molecules is greatly increased. We will see in Section 4 that this occurrence is produced by the presence of water, whose essential role is usually neglected in the biological modelling. This crucial role of water in intermolecular interaction is also neglected in Cosic's model. As a matter of fact, molecular interactions within a biological organism are not taking place in the empty space, but they occur in an aqueous medium, since water, as we will point out in Section 4, is the main constituent of living matter. 

### 2.3. The Dicke Theory

The formation of “electromagnetic cavities” in living matter appears as an essential task for elucidating the biomolecular interaction dynamics. In modern physics the formation of coherent electromagnetic fields has been historically connected with the development of laser. As said above, the appearance of a coherent field occurs in the presence of an external supply of energy, a *pump* in the physical jargon. Moreover an externally supplied cavity, whose size allows us to select a specific wavelength of the coherent field, is needed. Coherence appears when suitable boundary conditions are met and only when the system is pumped. However this could not be the case for living systems where no pumps and cavities are provided from outside. A first contribution to the solution of this problem has been offered by Dicke [49, 50], whose theory justifies the occurrence of a large increase of the field energy (superradiance) together with the appearance of the typical quantum phenomenon known as stimulated emission radiation (superfluorescence). In the present paper we do not deal with the complete Dicke theory, but only with its application, proposed by Li, to the emergence of coherence without an externally provided cavity. Li's model is based on the radiation theory advanced by Dicke in 1954, according to which, for distances between two emitters smaller than the wavelength of the light emitted, the emission must happen cooperatively, and the field between the emitters cannot be random but has to be coherent. The better this “Dicke condition” is fulfilled, the more the coherence increases. The emitters then cannot be considered as independent individuals, because they are embedded in and interacting with a common radiation field. Being coupled with the same e.m. field, they are part of a communicating system and are continuously getting information about each other. Moreover the coherent e.m. field is not emitted out of the ensemble of molecules to which it is coupled but is kept within it, so this case could be termed *subradiance*. This property will be a major feature of the quantum electrodynamics (QED) treatment given in Section 4. 

For the optical range of ultralow bioluminescence (biophotons), the Dicke condition is always fulfilled in biopolymers within the cell. DNA fulfills it optimally, as the distances between base pairs (3 Ångstrom) are much smaller than the wavelength of visible light (~5000 Å). For the volume of the cell, it is fulfilled for longer wavelengths such as microwaves.

Popp postulates that destructive and constructive interference between radiating sources of biological systems (biomolecules, organelles, cells, organs, organisms, and populations) may play a central role in biological organization [51]. Within the coherence volume between the emitting systems, the radiation may sustain a coherent interference field for considerable lengths of time. By the mechanism of destructive interference, which leads to the cancellation of force vectors, the system can trap photons and thereby store energy. On the other hand, by constructive interference, in the coherence volume between the radiators, stored photons are released. This is equivalent to the creation of a photochemical potential and constitutes a basic mechanism of energy transfer and of communication. The same mechanism also creates long-range attraction and repulsion forces in the coherence volume between identical systems, by which pattern formation and cell adhesion may be achieved. This force does not depend on electrical charges or magnetic moments, only on coherence length, the amount of stored energy, and the Planck constant. Its order of magnitude may amount to 10–12 *N* or even more. Li contends that the theory of Dicke is a major breakthrough in radiation theory as fundamental as Planck's and is universal for the discussion of all real systems including biological systems, whilst the realm of validity of Planck's theory is an ensemble of independent radiators in thermal equilibrium [43]. The nature of the interaction between particles that the conventional approach visualizes as a random, mechanical collision of isolated, hard ball-like particles thus is promoted to a highly ordered communicative and cooperative interconnectedness in a connecting field. 

As Dicke himself stated, his theory also is an alternative formulation of the laser concept. The usual technical approach of engineers treats “the laser as a feedback amplifier, the amplification being treated as resulting from stimulated emission, the upper energy state having an excess population. The second approach treats the laser not as an amplifier, but rather as a source of spontaneous emission of radiation with the emission process taking place coherently” [50].

The Dicke theory meanwhile has found full experimental confirmation [52]. It is an important predecessor of the quantum field approach we will present in Section 4, in that it first suggested how the electromagnetic field can be trapped within matter, thereby forming a novel form of matter-field unity and giving rise to a laser-like process. However, it still considers the collective interaction as an ensemble of two-body interactions and therefore cannot fully account for the embedded and interactive nature of all living systems. 

## 3. Supramolecular Approaches to Communication and the Emergence of Order in Biology

In the first half of the 20th century, a number of holistic approaches to biology, based on long-range correlations between molecules and cells, have been developed, mainly in developmental biology [15, 16]. They were part of a countermovement to the reductionist program of the “Berlin school” of physically oriented physiologists, among them Emil DuBois-Reymond and Hermann von Helmholtz, whose work laid the foundations for molecular biology and scientific medicine. The development was initiated by the controversy between Wilhelm Roux and Hans Driesch about their embryological experiments with frog and urchin eggs in the late 1880s and early 1890s, where Driesch showed that, in the early stages of development, the embryo possessed the faculty of regulation, that is, was able to develop into a whole organism even when parts were damaged or destroyed. Driesch concluded that the state of each part of the organized whole of the organism was dynamically codetermined by the neighbouring parts, such that there is a relational structure between the parts that is specific for the organism. Biological function was dependent on the position within the whole, not on the mechanical preformation of parts, as Roux had assumed. Therefore Driesch countered Roux' mechanistic “mosaic theory of development” with his own theory of a vitalistic “entelechy,” a field-like factor responsible for organismic form and structure that he deemed not accessible to scientific investigation. 

Driesch's vitalistic challenge caused a fundamental crisis in developmental biology which led to a reformulation of basic concepts in experimental embryology and cell biology. At first, a number of outstanding biologists followed Driesch by assuming one of several kinds of neovitalistic stances, among them Jacob von Uexküll, John Scott Haldane, Constantin von Monakow, and Richard Semon, but by 1930 the movement culminated in a series of proposals for a nonvitalistic alternative to mechanistic biology under the name of holism or organicism. This group of approaches, far from being marginal, played a considerable role in European, American, and Russian biology from the 1920s to the 1950s. Inspired by the work of Spemann on “inducers” and “organizers” [53], a number of mainly British and American biologists, among them Paul Weiss, Joseph Needham, Ross Harrison, Conrad Waddington, and Alexander Gurwitsch, set out to identify the organizing principles in developing systems by careful analysis on the level of tissues [54, 55]. In the work of these scientists, the concept of a “biological field” or “morphogenetic field” emerged as a central metaphor for uniting both the organizer and the organized tissues and for understanding the integrating and organizing principles in organisms. Implicitly, the field properties of living organisms were already recognized in Driesch's 1891 suggestion that the whole embryo was a “harmonious equipotential system.” The field concept was introduced into biology by Gurwitsch [56] and by Weiss [57] who also first proposed to use the concept of “system” in biology in 1924. Weiss, who had trained as an engineer and was well grounded in the physical sciences, first used it to explain the results of his and others' work on limb regeneration in amphibians but later generalized it to ontogeny as a whole. In 1925–1930, he concluded from his work on bone regeneration that a “limb field” was directing differentiation in the amputated stump; it was a property of the field district as a whole and not of a particular discrete group of elements. In the 1940s his studies on nerve orientation in repair and growth processes led him to think that tissue behaves as a coherent unit. In the 1950s Weiss investigated chondrogenesis (cartilage formation) and specified the role of tissue environment in general and of mechanical stresses in particular in the differentiation of mesenchyme cells into cartilage. The observation that precartilaginous cells of different types developed in cell culture into the respective specific type of cartilage according to the *in vivo* pattern convinced him that they possessed highly specific fields with

  distinctive morphogenetic properties determining the particular pattern of cell grouping, proliferation, and deposition of ground substance which, in due course, lead to the development of a cartilage of a distinctive and typical shape [58].

Weiss referred to the ordering processes leading to the emergence of organ and tissue structuring during development as “field actions” but emphasized that this should not be taken as an explanation. He defined the biological field as

  a condition to which a living system owes its typical organization and its specific activities. These activities are specific in that they determine the character of the formations to which they give rise. (…) Inasmuch as the action of fields does produce spatial order, it becomes a postulate that the field factors themselves possess definite order. The three-dimensional heterogeneity of developing systems, that is, the fact that these systems have different properties in the three dimensions of space, must be referred to a three-dimensional organization and heteropolarity of the organizing fields [59].

The fields were specific that is, each species of organism had its own morphogenetic field. Within the organism, there were subsidiary fields within the overall field of the organism, thus producing a nested hierarchy of fields within fields.

The Russian embryologist and histologist Alexander G. Gurwitsch was the first to introduce the field concept into biology in 1922, under the name of “embryonal, or morphogenetic field” [60].

  The place of the embryonal formative process is a field (in the usage of the physicists) the boundaries of which, in general, do not coincide with those of the embryo but surpass them. Embryogenesis, in other words, comes to pass inside the fields (…). Thus what is given to us as a living system would consist of the visible embryo (or egg, resp.) and a field [56].

While Driesch's entelechy was of the “Aristotelian type” of biological fields [61], that is, organizing principles immanent to the organism, evolving with the organism, and playing a causal role in organizing the material systems under their influence, Gurwitsch's morphogenetic fields rather belong to the “Platonic type”; that is, they are eternal, changeless transcendent forms or ideas of essentially mathematical or geometric nature—“spatial, but immaterial factors of morphogenesis.” However, although inspired by Driesch, in contrast to the German scientist, Gurwitsch was determined to put the hypothesis of the biological field to the experimental test. In experiments first with onion roots and later with many other organisms, cells, and tissues, he discovered what he called “mitogenetic radiation” and today is known as “ultraweak photon emission” or “biophoton emission,” an ultraweak electromagnetic broad-band (200–800 nm) radiation emitted by practically all living organisms [51, 62, 63]. With his suggestion that the morphogenetic field produces a thermodynamic nonequilibrium in the molecular ensembles of cells and tissues and that, on the other hand, the potential energy released by the decomposition of such excited non-equilibrium molecular complexes can be transformed into kinetic energy leading to directed movement of substance, he became one of the early proponents of what is today known as dissipative structures. 

Although the field concepts of these early 20th century biologists referred to the model of physical, especially electromagnetic, fields, the organicists generally considered the biological field as a purely heuristic concept, a tool for understanding the phenomena of development, regeneration, and morphogenesis and for making predictions for experimental testing. Even those of them tending to the Aristotelian position usually preferred not to go beyond the analogy and to leave the exact nature of the fields open. The notion of real electrical, electromagnetic, or otherwise physical fields of long-range force carried too clearly vitalist implications for them. On the other hand, to their taste, the ideal, strongly geometrical fields of Gurwitsch were too dematerialized and too far from biochemical reality [54]. For Weiss [59], the field concept was also a mean to remain open as to the nature of the organizing factors as long as the tools for determining it were not adequate. It seems that at that time both, biology and physics, were not yet enough developed to accommodate such a possibility. In physics, there was neither enough experimental evidence for the existence of electromagnetic fields in living organisms nor for the biological effects of e.m. fields, and the implications of quantum theory for a field perspective of life were not yet recognized. 

Another reason for the late acceptance of the electromagnetic aspect of organisms was the success of the biochemical approach to life. Among the several reasons quoted in [64] for the fact that the concept of the biological field has almost completely vanished from biology in the 1950s, the most important one was the rise of genetics as an alternative program to explain development. In the 1930s the biological field had been a clear alternative to the gene as the basic unit of ontogeny and phylogeny, and this was seen as a threat to the rise of genetics as the leading field of biology. This was the reason why Thomas Hunt Morgan, one of the founders of modern genetics and himself originally a developmental biologist, actively fought the concept of the morphogenetic field and tried to denounce and ridicule it in all possible ways, although Morgan was not able to present any evidence against fields [64–66]. With the breakthrough of genetically oriented molecular biology through Watson and Crick's 1953 model of DNA, field theories were definitely out [63], and the predominance of the biochemical-molecular approach in biology began, as we know it today. 

However, today the situation has changed again. Since the early 1980s, the group of “structural biologists” around Brian C. Goodwin has advocated the concept of the biological field as an adequate basis for a unified theoretical biology [67–69]. They state that field properties of organisms underlie both reproduction and regeneration which share the essential feature that from a part a whole is generated [69]. Reproduction is not to be understood in terms of Weismann's germ plasm or DNA but rather as a process arising from field properties of the living state. They postulate a feedback loop between morphogenetic fields and gene activity: the fields generate ordered spatial heterogeneities that can influence gene activities; gene products, in turn, can influence the fields, destabilizing certain patterns and stabilizing others. More recently, a group of leading biologists, Gilbert, Opitz, and Raff, have challenged the adequacy of genetics for alone explaining evolution and the devaluation of morphology and have demanded the rehabilitation of the biological field [64, 66]. They suggest that evolutionary and developmental biology should be reunited by a new synthesis in which morphogenetic fields are proposed to mediate between genotype and phenotype and to be a major factor in ontogenetic and phylogenetic changes. Gene products should be seen as first interacting to create morphogenetic fields in order to have their effects; changes in these fields would then change the ways in which organisms develop. 

Serious progress in bioelectromagnetic field theory, although there were earlier precursors like Keller [70], Crile [71], Lund [72], and Lakhovsky [73], started not before the work of Harold S. Burr [74, 75] and Presman [37]. The concept of Burr and Northrop, based on Burr's work on bioelectric potentials, is summarized in the following quote:

  The pattern of organization of any biological system is established by a complex electro-dynamic field, which is in part determined by its atomic physicochemical components and which in part determines the behavior and orientation of those components. This field is electrical in the physical sense and by its properties it relates the entities of the biological system in a characteristic pattern and is itself in part a result of the existence of those entities. It determines and is determined by the components. More than establishing pattern, it must maintain pattern in the midst of a physicochemical flux. Therefore, it must regulate and control living things, it must be the mechanism the outcome of whose activity is “wholeness”, organization and continuity. The electro-dynamic field then is comparable to the entelechy of Driesch, the embryonic field of Spemann, the biological field of Weiss [74].

The groundbreaking work of Presman marks the beginning of modern e.m. field theories for biology [37]. He argued that environmental e.m. fields have played some, if not a central, role in the evolution of life and also are involved in the regulation of the vital activity of organisms. Living beings behave as specialized and highly sensitive antenna systems for diverse parameters of weak fields of the order of strength of the ambient natural ones. According to Presman, e.m. fields play an important role in the communication and coordination of physiological systems within living organisms and also mediate the interconnection between organisms and the environment. However, bioelectromagnetic field theories only recently have reached a certain maturity due to the general progress in electromagnetic theory, bioelectromagnetics, and particularly non-equilibrium thermodynamics and quantum theory [76]. Mainly this was achieved in the work of Herbert Froehlich and what could be called the Froehlich school of biophysics, including the Prague group of Pokorny and the Kiev group of Davydov and Brizhik, in the quantum field theoretical approach of Umezawa, Preparata, Del Giudice, and Vitiello and in the biophoton research of the Popp group in Germany. 

## 4. A Quantum Field Approach to Biological Dynamics and Biocommunication

Quantum Physics has emerged from a major paradigm shift with respect to Classical Physics which still provides the framework of the vision of nature of most scientists. This change of paradigm has not yet been completely grasped by contemporary science so that not all the implications of this change have been realized hitherto. The classical paradigm assumes that every physical object can be isolated from any external influence and can be studied independently of other objects. Since the system is isolated, the relevant physical variables can be unambiguously defined and assume specific values which remain constant in time; in this way we are able to derive a number of principles of conservation such as, for instance, of energy, momentum, and angular momentum. In this scheme change and movement can emerge only by a supply of adequate quantities of such variables from outside. Matter is consequently considered inert and can move only if acted upon by an external agent applying a force. 

Therefore physical reality is assumed to be a collection of separate objects, for instance, atoms, having an independent a priori existence, kept together by external forces whose existence demands a suitable space-time distribution of energy. The formation of an aggregate of atoms requires therefore an adequate supply of energy; the same requirement applies to every change of its configuration. The components of an aggregate are considered to have the same nature when they are isolated and when they are aggregated; the interaction is not changing their nature and remains somehow peripheral. 

The quantum paradigm not only gets rid of the concept of inert matter but also denies the possibility to simultaneously define all the variables of a physical system. Every physical object (either a material particle or a field) is conceived as intrinsically fluctuating; its definition should therefore involve a phase *φ*. It exhibits different aspects not simultaneously compatible when addressed from different points of view. This amounts to the choice of different subsets of mutually compatible variables to define the system and therefore to different representations of it. Physical variables are therefore grouped into families of compatible variables. Different families cannot be defined simultaneously so that the principle of complementarity holds that different families of variables, once defined, give rise to different pictures of the same object, not simultaneously compatible among them. 

An important outcome of Quantum Physics concerns the concept of localizability of an object. Quantum Physics supports the objection against localizability raised by Zeno of Elea who used to say that a flying arrow is not moving since it is at each moment of time in its own place. In this context, a role is played by the quite subtle concept of the quantum vacuum [20]. The strict definition of the vacuum is the minimum energy state of a physical system. The energy of the vacuum should be conceived as the energy necessary to maintain the bare existence of the system. However, since a quantum system cannot be ever at rest (in Quantum Physics there is a “horror quietis”, i.e. “fear of resting”), the vacuum should contain the energy related to the system's quantum fluctuations. However, quantum fluctuations cannot be observed directly, and their existence must be inferred from indirect evidence. Consequently, physicists are forced to formulate a principle of invariance of the Lagrangian (the mathematical function which gives rise to the equations of motion from which the actual behavior of the system is inferred) with respect to arbitrary changes of the local phase of the system (the so-called local phase invariance of the Lagrangian). In the following we describe its consequences in nonmathematical terms. The local phase invariance is shown to hold if a field exists which is connected to the space-time derivatives of the phase. In the case of a system made up of electrically charged components (nuclei and electrons of atoms), as, for instance, a biological system, this is just the electromagnetic (e.m.) potential **A**_*μ*_, where *μ* is the index denoting the four space-time coordinates *x*_0_ = *ct*, *x*_1_, *x*_2_, *x*_3_. The electric and magnetic fields are suitable combinations of the space-time derivatives of **A**_*μ*_. In order to get the local phase invariance, we should assume that the Lagrangian is invariant with respect to specific changes of the field **A**_*μ*_. Thus a specific principle of invariance, named “gauge invariance,” emerges; hence the name “gauge field” denotes **A**_*μ*_. Actually it is well known that the Maxwell equations just obey the gauge invariance, which in Quantum Physics becomes the natural partner of the phase invariance to produce our world; quantum fluctuations give rise to e.m. potentials which spread the phase fluctuations beyond the system at the phase velocity. This gives an intrinsic nonlocalizability to the system and prevents a direct observation of quantum fluctuations. Through the e.m. potential, the system gets a chance to communicate with other systems. Notice that all e.m. interactions occur in a two-level way; the potential keeps the interacting particles phase-correlated whereas the combination of its space-time derivatives, named e.m. field, accounts for the forces involved. The lower level, the potential, becomes physically observable only when the phase of the system assumes a precise value. The structure of electrodynamics makes possible the presence of a potential also when both electric and magnetic fields are absent, whereas on the contrary fields are always accompanied by potentials. 

The above solution which stems from the mathematical formalism of Quantum Field Theory (QFT) [5] opens the possibility of tuning the fluctuations of a plurality of systems, producing therefore their cooperative behavior. However, some conditions must be met in order to implement such a possibility. Let us, first of all, realize that in Quantum Physics the existence of gauge fields, such as the e.m. potential, dictated by the physical requirement that the quantum fluctuations of atoms should not be observable directly, prevents the possibility of having isolated bodies. For this reason, the description of a physical system is given in terms of a matter field, which is the space-time distribution of atoms/molecules, coupled to the gauge field with the possible supplement of other fields describing the nonelectromagnetic interactions, such as the chemical forces. 

In Quantum Physics, the space-time distribution of matter and energy has a coarse-grained structure which allows its representation as an ensemble of quanta (particle representation). However, according to the principle of complementarity, there is also another representation where the phase assumes a precise value; this representation which focuses on the wave-like features of the system cannot be assumed simultaneously with the particle representation. The relation between these two representations is expressed by the uncertainty relation, similar to the Heisenberg relation between position and momentum,

(1) $$\begin{array}{c} \delta N\delta \varphi \geq \frac{1}{2} \end{array}$$

connecting the uncertainty *δN* of the number of quanta (particle structure of the system) and the uncertainty *δφ* of the phase (which describes the rhythm of fluctuation of the system). 

Consequently, the two representations we have introduced above correspond to the two extreme cases.

1. If *δN* = 0, the number of quanta is well defined, so that we obtain an atomistic description of the system but lose the information on its capability to fluctuate, since *δφ* becomes infinite. This choice corresponds to the usual description of objects in terms of the component atoms/molecules.
2. If *δφ* = 0, the phase is well defined, so that we obtain a description of the movement of the system but lose the information on its particle-like features which become undefined since *δN* becomes infinite. Such a system having a well-defined phase is termed coherent in the physical jargon.

In the phase representation, the deepest quantum features appear since the system becomes able to oscillate with a well-defined phase only when the number of its components becomes undefined, so that it is an open system and able to couple its own fluctuations to the fluctuations of the surroundings; in a sense, such a coherent system, like a biological one, is able to “feel” the environment through the e.m. potential created by its phase dynamics. 

In conclusion, a coherent system involves two kinds of interaction:

1. an interaction similar to that considered by Classical Physics, where objects interact by exchanging energy. These exchanges are connected with the appearance of forces. Since energy cannot travel faster than light, this interaction obeys the principle of causality;
2. an interaction where a common phase arises among different objects because of their coupling to the quantum fluctuations and hence to an e.m. potential. In this case there is no propagation of matter and/or energy taking place, and the components of the system “talk” to each other through the modulations of the phase field travelling at the phase velocity, which has no upper limit and can be larger than *c*.

### 4.1. Emergence of Coherence within an Ensemble of Microscopic Components

The process of the emergence of coherent structures out of a crowd of independent component particles has been investigated in the last decades and is presently quite well understood [3, 11]. Let us describe a simple case where a large number *N* of atoms of the same species, whose particle density is *N*/*V*, are present in the empty space in the absence of any externally applied field. However, according to the principles of Quantum Physics, one e.m. field should always be assumed to be present, namely, the field arising, as said above, from the quantum fluctuations of the vacuum. The presence of this field has received experimental corroboration by the discovery of the so-called “Lamb shift,” named after the Nobel prize winner Lamb [77]. He discovered as far back as in 1947 that the energy level of the electron orbiting around the proton in the hydrogen atom is slightly shifted (about one part per million) with respect to the value estimated when assuming that no e.m. field is present. Further corroboration for the existence of vacuum fluctuations is provided by the Casimir effect [78–80]. Therefore a weak e.m. field is always present, just the one arising from the vacuum quantum fluctuations. 

We give now a qualitative argument for the emergence of coherence [81]. Let us assume, for the sake of simplicity, that the atoms can exhibit just two configurations: the minimum energy state which is the vacuum of the atom and the excited state having an excitation energy *E* = *hν* (the Einstein relation connecting *E* to the frequency *ν* of a photon having the same energy, where *h* is the Planck constant). A quantum fluctuation having the same frequency *ν* and a corresponding wavelength *λ* = *c*/*ν*, where *c* is the speed of light, is therefore able to excite an atom present in the volume *V* = *λ* ^3^ of the fluctuation, that is, to raise it from the ground configuration to the excited configuration. The excited atom releases the excitation energy and comes back to the ground configuration after a typical time dictated by atomic physics, that is, the decay time of the excited state. 

We should now pay attention to an important mismatch of the scales present in the problem we are dealing with. An atom has a size of about 1 Ångstrom (Å) which amounts to 10^−8^ cm, whereas a typical excitation energy is in the order of some electron volts (eV's), corresponding to a wavelength of the associated e.m. fluctuation in the order of some thousand Ångstroms. This means that the tool (the e.m. fluctuation) able to induce a change of configuration in the atom is some thousands of times wider than the atom itself. Hence a single quantum fluctuation can simultaneously involve many atoms; in the case, for instance, of the water vapor at boiling temperature and normal pressure, the exciting e.m. mode (in this case 12 eV) would include in its volume 20,000 molecules. Let us assume now that in the volume *V* = *λ* ^3^ of the fluctuation there are *N* atoms. Let *P* be the probability (calculated by using “Lamb shift”—like phenomena) that an isolated atom is excited by an e.m. quantum fluctuation. Therefore the probability *P*_*N*_ that one out of the *N* atoms gets excited by the fluctuation is

(2) $$\begin{array}{c} P_{N}=PN=P\lambda^{3}\left(\frac{N}{V}\right)=P\lambda^{3}d, \end{array}$$

where *d* is the density of atoms. We can see that there is a critical density *d*_crit_ such that *P*_*N*_ = 1, which means that the fluctuation excites *with certainty* one atom. In such conditions, the virtual photon coming out from the vacuum is “handed over” from one atom to another and gets permanently entrapped within the ensemble of atoms, being busy in keeping always at least one atom excited; according to this dynamics atoms acquire an oscillatory movement between their two configurations. In a short time, many quantum fluctuations pile up in the ensemble, producing eventually a large field which keeps all atoms oscillating between their two configurations. Moreover, the field gets self-trapped in the ensemble of atoms since its frequency becomes smaller; actually the period of oscillation *T* of the free field should be extended by adding the time spent within the excited atoms. Consequently, the frequency *ν* = 1/*T* decreases. As a further consequence, the mass *m* of the photon, which is zero in the case of a free field, becomes imaginary. In fact, by using the Einstein equation for the photon mass and energy, we get

(3) $$\begin{array}{c} m^{2}=E^{2}-c^{2}p^{2}=h^{2}\left(\nu^{2}-\frac{c^{2}}{\lambda^{2}}\right)\leq h^{2}\left(\nu_{\text{free}}-\frac{c^{2}}{\lambda^{2}}\right)=0. \end{array}$$

The fact that *ν* ≤ *ν*_free_ implies that the squared mass *m* ^2^ of the trapped photon becomes negative and the mass *m* imaginary, which implies furthermore that the photon is no longer a particle and cannot propagate. The field generated within the ensemble of atoms cannot be irradiated outwards and keeps the atoms oscillating up and down between the two configurations. Finally, in this way the ensemble of atoms becomes a self-produced cavity in which the e.m. mode is trapped, and, like in the cavity of a laser, the field becomes coherent, that is, acquires a well-defined phase, in tune with the oscillations of the atoms, which therefore become coherent, too. The more realistic case of atoms having a plurality of excited states has been also successfully addressed and needs a more sophisticated mathematics [3]. Among all the excited levels, the one selected for giving rise to the coherent oscillation is the level requiring the smallest time to self-produce a cavity. 

The region becomes a coherence domain (CD) whose size is the wavelength of the e.m. mode, where all atoms have tuned their individual fluctuations to each other and to the oscillation of the trapped field. The size of the coherence domain cannot be arbitrary but is determined in a self-consistent way by the dynamics underlying the emergence of coherence via the wavelength of the involved e.m. mode. A coherent system is therefore an ensemble of self-determined e.m. cavities. The fact that a biological system appears to be a nested ensemble of cavities within cavities of different sizes (organs, tissues, cells, organelles, etc.) having well-defined sizes is a strong indication for its coherence. In a CD there is a common phase, specific of the CD, which is therefore an object governed by a dynamics which eliminates the independence of the individual components and creates a unitarily correlated behavior of all of them, governed by the e.m. field. It is interesting to note that the German botanist Julius Sachs, as far back as in 1892, coined the term “energide” to denote the unity of matter and field constituting the nature of cells, namely, the unity of matter and the so-called “vital force” which gives to biological matter its special active properties discriminating it from inert nonliving matter [82]. 

Communication within the domain takes place at the phase velocity, since the messenger is the e.m. potential and not the e.m. field. Therefore it is much faster than communication implying energy and/or matter propagation. Moreover, the correlation can involve only atoms/molecules able to oscillate at the same frequency or some harmonics thereof (resonance). Finally, the emergence of coherence is a spontaneous event once the critical density *d*_crit_ = (*N*/*V*) is exceeded. 

The above argument has not taken into account temperature and thermal effects, which are limiting constraints for coherence generation. When we include the thermal motion of atoms and the related thermal collisions, we realize that a fraction of the coherent atoms will be pushed out of tune by the collisions, so that above the critical temperature where this fraction becomes the unity, the coherent state cannot exist anymore [3].

In conclusion, an ensemble of microscopic units (atoms, molecules, etc.), provided that they are composite systems having a plurality of internal configurations, becomes always spontaneously coherent once a critical density is overcome and temperature is lower than a critical threshold. 

The coherent dynamics outlined above produces stable coherence domains since the coherent state is a minimum energy state, where the energy is lower than the energy of the original ensemble of noncoherent independent particles, because of the interaction between the atoms/molecules and the self-trapped e.m. field. Therefore the coherent system cannot decay spontaneously into another state having a still lower energy. The coherent state can be dismantled only by a supply of external energy large enough to overcome the “energy gap,” that is, the difference of energy between the coherent state and the noncoherent ensemble of its components before the onset of coherence [20]. The energy gap therefore protects both the integrity and stability of the coherent system and the individual integrity of the components against (small) external disturbances. 

However, the examples of coherent systems that are more widely known in common scientific culture, for example, lasers, occur in the presence of an external supply of energy, which in the physical jargon is termed a “pump.” Coherence in these cases appears only when the system is pumped, so that it cannot arise and survive spontaneously, and the system is faced by the limiting element of the inverse process, decoherence. Consequently living systems cannot be properly considered as lasers in the conventional sense. The analogy of living organisms with lasers should therefore be considered as a metaphor [3].

### 4.2. The Special Case of Liquid Water

In the present context, a decisive role is ascribed to water, as this is the case in the living organism. Therefore let us now treat the special case of the formation of coherence domains in this life-sustaining substance, which has been examined in depth and which allows us also to come back to the coherent dynamics envisaged above [3, 81, 83–86]. The emergence of coherence, as described above, accounts successfully for the vapor-liquid phase transition and for the thermodynamics of water. A quantitative agreement with experimental evidence has been found [87, 88]. 

Moreover, a peculiar feature appears in the case of water. The coherent oscillation of the water molecules, which induces the formation of the coherence domains, occurs between the molecule's ground state and an excited state at 12.06 eV, which is slightly below the ionization threshold at 12.60 eV. The electron cloud of the water molecule oscillates between a configuration where all electrons are tightly bound (in this configuration water is an insulator and a mild chemical oxidant, since it is able to bind an extra electron) and a configuration where one electron is almost free (in this configuration water becomes a semiconductor and a chemical reducer, since it is able to release electrons). We have therefore the following consequences.

1. Within the CD a coherent plasma of quasifree electrons is present; the coherence of the plasma is the consequence of the coherence of molecules. This plasma is able to give rise to coherent “cold” vortices, since coherence prevents collisions among electrons which would occur, should electrons be independent, when external energy is supplied. In this way the water CD acquires a spectrum of coherent excited states [89]. For water CD's we can iterate the argument given above for atoms/molecules and show that water CD's can become coherent among them, giving rise to a “supercoherence” [84]. We get, in the case of water, a hierarchy of levels of coherence which starts from the elementary domains, whose size is 0.1 microns (which is the wavelength corresponding to an e.m. mode of 12.06 eV) and reaches much higher scales corresponding to “superdomains” as wide as microns (cells), centimeters (organs), and meters (organisms), parallel to the corresponding hierarchy found in biology. This hierarchy underlies the fractal nature of living organisms; it has been recently proven that fractals are just the consequence of the sequence of nested coherent dynamics [90].
2. Water CD's are able to release electrons, either by quantum tunneling or by mild external excitations. The pair coherent water-noncoherent water (as it is present at the boundary of a domain) is therefore a redox pile able to supply the electrons needed in the redox chemical processes which produce the bulk of the biological energy. Szent-Gyorgyi has long ago shown that electrons should be available on biological surfaces [22, 31, 91]. A difference of electric potential in the order of several tens of millivolts (the same order as the membrane potential) has been estimated to be present across the interface at the boundary of water CD's [85].

In conclusion, liquid water (which contributes about 70% of the total mass and 99% of the total number of component molecules of a living organism) exhibits a twofold inner dynamics. 

(1) Through the hierarchical scale of nested coherence domains just described, water is able to store energy in the coherent electron vortices in the CD's whose lifetime can be very long because of coherence [89]; the long lifetime of the single excitations enables a pile-up of excitations which are unified by coherence into single excitations of higher energy [7], producing a sharp decrease of entropy. This feature confirms the proposal of Schrodinger [92] about the need of negative entropy (negentropy) for the appearance of order in living systems. The water CD appears as a device able to collect energy from the environment as an ensemble of many independent low-energy excitations (high entropy) and to transform them into a single excitation of high energy (low entropy) [8]. This property makes water CD's likely candidates to be dissipative structures à la Prigogine [10]. Let us elucidate better this last point. Appearance of coherence in a physical system implies a spontaneous decrease of its entropy, since energy gets concentrated to a smaller set of degrees of freedom than before the onset of coherence. This phenomenon is possible without violating the second law of thermodynamics only if the system is open; the release of energy outwards, with the connected increase of entropy of the environment, is just the condition for the decrease of the internal entropy of the system. Consequently a coherent system is necessarily an open system. Moreover the spontaneous decrease of its internal entropy transforms its total energy into free energy able to perform external work. This is just the definition of a dissipative structure [7, 8]. When the energy stored in a CD changes the frequency of the CD so that it becomes equal to the frequency of one nonaqueous molecule present in the surroundings, this molecule can be accepted as a further partner of the CD and receives by resonance the stored energy, getting chemically activated and able to react chemically. The energy released in the chemical reaction is in turn taken over by the water CD, which correspondingly changes again its own frequency and becomes able to attract different molecular species. The range of attraction between CD's and coresonating molecules has the size of the CD and then provides the long-range selective attraction among molecules that is sorely missing in modern biochemistry [8, 81]. 

The above consideration allows us to sketch a first rough scheme of a picture of biochemical processes not based on diffusion and random molecular movements, where molecular encounters are not random but obey specific “organic codes” evolving in time and where each step of the biochemical sequence is determined by the previous one. In the dynamics outlined here energy plays the major role. 

(2) There is, however, a second dynamics always coupled with the first one, which plays a major role in the communication at a long distance. A system is coherent since its phase has a well-defined value. An ensemble of nested time-dependent coherence domains, such as those possible in aqueous systems (living organisms are of course aqueous systems), exhibits therefore an extended phase field evolving in time. Within this field, communication travels faster than light, providing a connection at a large distance among the parts. According to the concepts described in the first part of this section, the variations of the phase produced by the dynamics of the biochemical scheme discussed above are carried within the larger coherent region by the e.m. potential which at each point triggers changes in the molecular dynamics (recall that the potential appears in the Schrodinger equation of the molecule). In this scheme the information gets spread around by an agent, the phase, able to travel faster than light, provided that the journey takes place within the coherent region, but no actual propagation of energy occurs. The energetic requirements of the flow of information are met solely by the energy present locally. In other words, the sequence of events is as follows: an energetic event (e.g., a chemical reaction) occurs at some place of the coherent region, its output energy induces a change of the phase of the host CD, and an e.m. potential then arises which reaches with a speed faster than light far away regions whose phase is correspondingly changed, implying a change of the local molecular dynamics. An actual transfer of energy and/or matter does not take place at any point in this sequence of events, removing therefore the usual objection raised against the real existence of phenomena which seem to imply the existence of an instantaneous action at a distance by living organisms [93]. 

The existence of a coupling among components of a living organism mediated by the e.m. potential could also provide a surprising unexpected rationale for the seeming paradox of large biological effects induced by the application of very weak electromagnetic fields. This occurs, for instance, when cell cultures are irradiated by low-intensity microwaves [94]. Cells exhibit large biological effects when the microwave irradiation occurs at selected values of the microwave frequency, according to a spectrum specific of the cell species. Above a very tiny threshold, the effect does not depend on intensity, provided that the intensity is below the value where thermal effects begin to be significant. This nonthermal effect of radiation coupled to the seeming paradox of effects disproportional to the amount of supplied energy could be understood by assuming that the effective agent is not the field but the potential. Further information on the effect of potentials on biological targets will be provided in Section 5 when we will discuss the experimental corroboration for the present theoretical scheme. 

### 4.3. Normal Water and Biological Water

We should now come back to the properties of the usual normal water, by realizing that the above complex dynamics with the interplay between chemistry and coherent quantum electrodynamics cannot occur in normal bulk water, that is, water far from surfaces. The reason is that in normal bulk water the interplay between electrodynamic processes inducing coherence and thermal processes hampering coherence gives rise to a continuously changing dynamic equilibrium where each molecule crosses very swiftly from a coherent to a noncoherent state, and vice versa. In such a situation the total number of molecules belonging to the two fractions, coherent and non-coherent, is constant at each temperature, as it occurs in the well-known case of superfluid liquid Helium [95]. However, the molecules actually belonging to the two fractions are continuously crossing over between the two fractions, producing a flickering landscape at a microscopic level. Since every observation implies a time average of the observed features on the time necessary for the observation, this explains why liquid water (and the other liquids too) looks homogenous and not like the two-phase liquid it really is. Consequently, a water CD does not live long enough to exhibit its long-time features and to produce a history. However, close to a surface, a wall, or a molecular backbone able to attract water, the attraction energy adds up to the “energy gap” produced by the coherent dynamics and gives rise to a better shielding against thermal disruption. As a consequence, interfacial water is almost all coherent. Within a living organism there is no point of the aqueous medium further removed from some surface or macromolecular chain than a fraction of a micron, so that we can infer that almost the whole biological water is interfacial and therefore coherent. 

The above property accounts for the observation that water in the hydration shells of biomolecules exhibits quite different properties than usual bulk water. In particular, it has been observed that this water looks like water at subzero temperatures (overcooled water), which resembles more and more to a glass when lowering temperature. This property has been investigated in the QED framework by Buzzacchi et al. [87] who have shown that the coherent fraction of water increases at decreasing temperature and coincides with the whole mass of the liquid at temperature around 200 K. At a temperature of 250 K coherent fraction has already a very high value, about 0.7. It is not strange that when coherent fraction gets increased by the interaction with a surface water looks like overcooled water. This property has been observed by many authors as, for instance, Levstik et al. [96]. Let us quote *verbatim* the statement of Pagnotta and Bruni [97]:

  interfacial and intracellular water is directly involved in the formation of amorphous matrices, with glass-like structural and dynamical properties. We propose that this glassiness of water, geometrically confined by the presence of solid intracellular surfaces, is a key characteristic that has been exploited by Nature in setting up a mechanism able to match the quite different time scales of protein and solvent dynamics, namely to slow down fast solvent dynamics to make it overlap with the much slower protein turnover times in order to sustain biological functions. Additionally and equally important, the same mechanism can be used to completely stop or slow down biological processes, as a protection against extreme conditions such as low temperature or dehydration.

A more impressive consequence of the interaction between liquid water and solid surfaces has been provided by the group led by Pollack [98–101]. They have found that the water layer close to a hydrophilic surface assumes peculiar properties. This layer may be as thick as some hundreds of microns and

- excludes all solutes; hence the name exclusion zone (EZ) is given to such layer;
- is about tenfold more viscous than normal water, looking like a glass;
- becomes fluorescent for wavelengths of 270 nm;
- assumes a surface charge of the same sign of the solid surface;
- exhibits a difference of electric potential with respect to the neighbouring bulk water of about 100 mV.

All these properties have been accounted for in the QED framework in a recent paper [102]. 

The presence of electromagnetic fields in the water layers close to the surface produces the appearance of a nondiffusive regime for particles present in these layers or on theirs surfaces. As a matter of fact, the presence of superdiffusive chemical signalling has been revealed on microfluidic chips [103]. Moreover the role of this nondiffusive regime near surfaces has been detected in the Belousov-Zhabotinsky (BZ) systems, where the self-ordering disappears when the amount of water decreases below a threshold [85]. 

The large size of the layers of EZ water (up to 500 *μ*m) is much larger than the size of CDs of water molecules (0.1 *μ*m) calculated in [3, 86], showing that the phenomenon of coherence among coherence domains as suggested by [84] is at work. The complex dynamics arising from the interplay of many scales of coherence which cannot occur in normal water therefore can arise near surfaces (recall the possible role of lagoons and clay surfaces in the origin of life; see [104, 105]) and can be safely at work within living organisms, suggesting the possibility of understanding at last their deepest dynamics. 

## 5. Experimental Probes of the Coherent Picture of Living Matter

The theoretical framework outlined above has increasingly received support by a growing body of evidence. First of all, one should realize that the QFT picture satisfies the two main requirements demanded by biological evidence we have discussed in the introduction: the existence of selective recognition and attraction among biomolecules (organic codes) and long-range connections among biocomponents which cannot be accounted for by the very short-range interactions implied by a purely chemical dynamics. 

However, we are now confronted by more direct evidence. In the last decade, the investigation of photosynthetic systems has produced evidence for the existence of coherent long-range connections between biomolecules. Let us quote verbatim from the abstract of a recent paper published in *Nature* [19]:

  Intriguingly, recent work has documented that light-absorbing molecules in some photosynthetic proteins capture and transfer energy according to quantum-mechanical probability laws instead of classical laws at temperatures up to 180 K. This contrasts with the long-held view that long-range quantum coherence between molecules cannot be sustained in complex biological systems, even at low temperatures. Here we present two-dimensional photon echo spectroscopy measurements on two evolutionarily related light-harvesting proteins isolated from marine cryptophyte algae, which reveal exceptionally long-lasting excitation oscillations with distinct correlations and anti-correlations even at ambient temperature. These observations provide compelling evidence for quantum coherent sharing of electronic excitation across the 5 nm wide proteins under biologically relevant conditions, suggesting that distant molecules within the photosynthetic proteins are “wired” together by quantum coherence for more efficient light-harvesting in cryptophyte marine algae.

The amount of pioneering research done on photosynthetic systems (see also the references of the quoted paper) opens the way to extended research on other biological systems in order to test the assumption that a coherent connection among biomolecules could be the general rule in biology. 

A direct corroboration of the electromagnetic nature of the biological dynamics and of the central role played by water in it has been provided by some recent work performed by a group led by the Nobel prize winner Montagnier et al. [18], which has been interpreted in the theoretical framework illustrated by the present paper [106]. Let us give a short summary of Montagnier's experiment.

Bacterial DNA sequences are suspended in pure bidistilled deionized water, and subsequently water is added increasing the dilution of the DNA sequences. The cuvette containing the suspension is put within a coil connected with a signal amplifier. Above a threshold of dilution, low-frequency e.m. fields (500–3000 Hz) are found at the condition that a very low-frequency (a few Hz) e.m. noise is present in the ambient. When the noise is absent, no signals are detected. The same occurs when the amount of water is below the critical threshold. The ensemble of these two circumstances suggests that water interacting with the DNA is responsible for the emission of fields and that, as illustrated in the last section, the energy of the signals is provided by the ambient noise being stored in water. Additional evidence is given by the circumstance that by further diluting the solution the intensity of the signals gets enhanced. A further fundamental feature appearing in the experiment is obtained by supplying the emitted signals to pure water contained in another vessel for a suitable time. This second vessel can even be located very far away and receive the signal coded in the first vessel at a distance, removing therefore the possibility of molecular contamination between the two vessels. After adding PCR molecules (the basic chemical components of DNA) to the irradiated water, the original DNA sequences appear in the irradiated liquid. The experimental evidence is consistent with the outcome of our theoretical framework that the supplied field induces a space-time distribution of the phase (which induces in turn an e.m. potential) in the irradiated pure water. This in turn drives the formation of a coherent biological aggregate, once the necessary biomolecules are supplied. 

Montagnier experiment suggests that ambient e.m. signals (as a matter of fact the e.m. potential) emitted from living organisms or from cosmic sources could affect the genic structure of organisms and have therefore possibly an impact on the species evolution. The investigation of this possibility could contribute significantly to the theory of evolution. However, we do not have yet data on this topic.

The Montagnier experiments suggest a key role for the e.m. potential. Direct evidence for this is obtained by a number of experiments performed in the last years (see, e.g., [13, 17]). Smith observed significant clinical effects on patients after their exposition to the action of fields produced by coils wound around ferromagnetic toroids; in this case, the magnetic field is tightly trapped within the toroid and cannot leak out so that the only field which can be present outside the toroid is the magnetic vector potential. Hence the appearance of clinical effects on the exposed patients can be produced solely by the magnetic vector potential. Since Quantum Physics prescribes that a pure potential can affect coherent systems only, the Smith results provide evidence suggesting that the living organism is a coherent system. More compelling evidence is provided by the Trukhan and Anosov experiment which does not involve human subjects. In their experiment the probed system is an ensemble of red blood cells suspended in water, and the measured variable is the velocity of sedimentation. The magnetic vector potential is switched on and off by switching on and off the electric current in the coil wound around the toroid. A significant change of the velocity of sedimentation of the erythrocytes is observed when the potential is present. Again, we obtain evidence that the affected biological targets are coherent systems and that a physical variable as the e.m. potential can become a biological agent. 

A last body of evidence comes from the appearance of new properties of liquid water when undergoing a special physical treatment. An Israeli group [107] has been able to observe a significant change of the physical properties of an aqueous system after irradiation by microwaves; they termed this specially treated water “neowater.” They observed that the space structure of the electrochemical deposits, left on the electrodes when using the neowater as a solvent, was much different from that left in the case of normal water, suggesting a different space distribution of the fields present in the solvent. Moreover, neowater gave different responses to physical probes such as NMR. The same properties were induced in water by using the suspension of microspheres of barium titanate or by the addition of fullerene molecules as a treating agent instead of microwaves. This is a surprising result since the same outcome has been produced by three completely different physical treatments. In our picture this would correspond to three different ways of enhancing the same feature, that is, the coherence of water. An intriguing feature appearing in neowater is the presence of a spatially ordered, crystal-like stable array of gas micro-bubbles. In normal water microbubbles are flickering in a random way. In our theoretical scheme, since nonaqueous molecules are expelled from within the coherence domains (because they are unable to resonate with water CD's), a bubble is going to appear in the interstice between a CD and its next neighbouring domain. The ordered array of microbubbles is therefore the consequence of the existence of an ordered array of water CD's, as it occurs when CD's become coherent among them (supercoherence). Since supercoherence can occur in a permanent way, as pointed out in Section 3, in interfacial water and biological fluids, neowater is a liquid where the possibility of supercoherence is extended to the bulk of the water by the physical treatment. Neowater should therefore be assumed to be intermediate between normal water and biological water. Further understanding of this property is provided by Giudice and Tedeschi [84]. They show that, by suspending triturated vegetal leaves and algae in water, after a suitable physical treatment, properties similar to those exhibited by neowater appear. The trituration of the suspended vegetals is meant to induce a highly enhanced coherent activity by them, because their “instinct of survival” induced by the “irritation” is assumed to increase their coherent activity in a strong way. The e.m. fields connected with this activity would then, as in the Montagnier experiment, imprint the surrounding water and produce coherence among coherence domains, namely, supercoherence. 

## 6. Outlook: Toward an Interplay between Physics and Biology

The quantum field theoretical picture, outlined in the present paper which has already got some experimental corroboration, opens new perspectives for future research. The feasibility of such a point of view has been already anticipated in some discussion on the origin of interconnectedness in biology [20]. A holistic picture of the living organism emerges which combines all the achievements reached by modern molecular biology with the collective dynamics made possible by the achievements of modern Quantum Physics. In each biological cycle, biomolecules encounter each other and behave exactly as in the ways discovered by molecular biology. However, molecular encounters do not occur through random diffusion movements but are driven by extended e.m. fields arising from the collective dynamics of water whose decisive role in the biological dynamics has been at last recognized. Consequently, a living organism cannot be conceived any longer as a mere collection of independent molecules mutually coupled by chemical interactions only but must be seen as a coherent ensemble, a matter field, whose evolution is driven by long-range e.m. fields whose features are in turn determined by the output of the chemical reactions. Biological dynamics is thus emerging from a close interplay of electrodynamics and chemistry. The ensuing picture of a living organism becomes that of a hologram, as suggested by Pribram for the brain [108]. 

The corresponding complex dynamics could be summarized as follows: water molecules, which account for the vast majority of molecular components of the organism, organize themselves into extended coherence domains where e.m. fields having a well-defined frequency are trapped inside. These coherence domains are able to collect chaotic energy (high entropy) from the environment and store it in the form of coherent excitations (low entropy) which change the frequency of the trapped e.m. fields. When this frequency matches the frequency of some nonaqueous molecules present in the surroundings, those molecules are attracted to the boundaries of the water CD's, coating them and possibly producing membranes [109]. The attracting forces are of the same type as the forces arising on the boundaries of laser beams and are the consequence of the following theorem: two particles able to oscillate at the respective frequencies *ν*_1_ and *ν*_2_ when immersed in an extended e.m. field oscillating at a frequency *ν*_0_, develop an attracting force (dispersive force), whose range coincides with the size of the background field. The attractive force becomes very large when the three frequencies *ν*_0_, *ν*_1_, and *ν*_2_ do not differ more than the thermal noise *kT*. The above theorem puts a constraint on which molecular species can be involved in the above dynamics. A molecule is able to participate in the coherent dynamics driven by water if and only if a frequency *ν*_*i*_ is present in its spectrum such that

(4) $$\begin{array}{c} h\left|\nu_{i}-\nu_{\text{CD}}\right|\leq kT. \end{array}$$

When molecules satisfy the constraint put by equation (4), they can steal energy from the thermal noise in order to resonate with the frequency of the CD and thus be involved in the coherent dynamics. Notice that the frequencies of oscillation of water CD's have been estimated [88] to be in the infrared range (0.2 ÷ 0.3 eV), so that the relevant part of the spectra of biomolecules is just the infrared one. 

In conclusion, in order to become a biomolecule, a given molecule is required to have in its spectrum a frequency contained in the range of the values of frequencies the water CD can assume. On this base the validity of the present scheme could be subjected to a test. Notice that, out of the about 100 amino acids known to chemists, only 20 are present in the biochemistry of living organisms. Here, the question must be asked why the other 80 amino acids are neglected by the organism. 

The occurrence of chemical reactions and the corresponding release of energy, as we have seen in Section 4, changes the frequency of the water CD and consequently also changes the involved molecular species; in such a way, we get finally a time-dependent scheme of molecular recognition and recall. Future research will have to work out testable schemes of biochemical reaction sequences, based on a detailed knowledge of infrared molecular spectra. 

All the events occurring within the coherent region are mutually coupled by the e.m. potential, so that they affect each other in such a way that we do not have linear sequences of local events but sequences where each step is a synchronous ensemble of coordinated events spanning the whole region. This feature is the basis of the holistic character of the living organism. 

The above concept applies in particular to the growth of the organism. The growing organism attracts new molecules through the field already formed within itself, so that the attraction does not occur at random but according to a code provided by the ensemble of frequencies present in the field. This attraction is a long-range attraction, since the field is falling off at the CD interface, as it occurs in all self-trapping cavities, and produces an exponentially decreasing evanescent tail. It is just this tail that allows the coherent system to explore its surroundings. In this way the evanescent tail could give rise to a morphogenetic field. These features of the evanescent field as responsible for the attraction of molecules on the CD surface account for the astonishing lack of biochemical mistakes, unavoidable within a random dynamics, and for the rapidity of the growth process which is well known to occur in a highly coordinated fashion. Ho and her colleagues have already proposed in 1994 that the morphogenetic field governing the growth of an embryo coincides with an e.m. potential [14]. A fascinating survey of morphogenesis has been given by [110]. A highly interesting contribution to this line of thinking has been given recently by Pietak [111]. However a detailed study of morphogenesis has not yet been performed and is a topic for future investigations.

The necessity of a resonance between biomolecules and water is at the origin of the appearance of geometrical shapes in living organisms. The frequency of oscillation of biomolecules should coincide with the frequency of oscillation of water CD's; however, the frequency of biomolecules depends on their chemical or other short-range bindings with neighbouring molecules. These bindings depend in turn on the mutual position of the interacting partners. On the contrary, the frequency of oscillation of CD's is not dependent on these local features. As a consequence, in order to match the frequency of the CD, biomolecules are compelled to assume those well-defined positions in space which allow them to oscillate at the same frequency as the CD's, hence the appearance of specific geometrical shapes in the structure of living organisms. The time dependence of the coherent dynamics implies a time dependence of the geometrical shapes, and this property could account for the interesting findings of Claverie and Jona Lasinio [112] about molecular structures. 

The concept of coherence applied here to biology has found so far a large scale application in the studies on brain dynamics [113–119]. According to this body of investigations, the activity of the brain is not the result of the activity of single neurons but emerges from the “mass action” (as Freeman has termed this collective activity) of macroscopic regions of the brain. A further offspring of this study is an approach to the problem of the emergence of consciousness [115, 116], which arises just from the permanent interaction of the brain with some part of the environment, that is, the ensemble of external oscillators resonating with the brain oscillators; Vitiello terms the external ensemble of oscillators “the Double” of the brain [116]. 

A final topic we address in this concise outlook concerns the concept of health. A healthy organism, according to the present scheme, should be a system where all the physiological and psychological subsystems are optimally synchronized and coherent with each other in a highly coordinated way [120]. Within this systemic state of coherence which is maintained by the communication through the e.m. potentials described above, the coherent interplay between fields and matter plays a decisive role. Each e.m. frequency must find a molecule able to respond to it, and each molecule present in the coherent region is governed by a corresponding field resonating with the respective molecular frequency. This could be a possible root of the well-known principle of biological homeostasis. 

It is entirely possible that the well-studied chemical effects of drugs could in actual practice be supplemented by electromagnetic effects induced by their presence. For instance, oligoelements are known to be essential for the health of human organisms. However, their actual concentration in the body is so low (10^−5^ moL) to make the assumption unlikely that their effect is the consequence of their presence on specific sites. A likely explanation, which of course demands experimental testing, could be that the atoms of an oligoelement do not act as chemical subjects but as e.m. antennae; these atoms could be conceived as the elements of a chain of e.m. stations which repeat and possibly amplify the e.m. signal ensuring its long-range transmission. The above speculation would suggest the existence, besides the local chemical effect, of an action at a distance of selected molecular species of drugs. 

The identity and stability of the coherent system are protected by the energy gap which is the amount of energy necessary to destroy coherence. Actually, a change of the structure of some components of a coherent ensemble by varying their oscillation frequency would put them out of tune with the general oscillation and hamper coherence. However, these local changes of the structure of the components would demand a supply of energy larger than the energy gap. Actually, local chemical reactions or other kinds of local processes could possibly release amounts of energy larger than the energy gap; for example, this release of energy could come from the local interaction of a microorganism hosted in the organism and some microscopic components of a larger coherent unit of the organism. In the case of an energy output of an interaction larger than the energy gap of the components, these last ones would be pulled out of coherence, damaging the wholeness of the organism. In this case the microorganism would turn into a pathogen, and illness would set in. In the conventional perspective, the therapy demands the killing of the pathogen, that is, a specific local therapy. However, the scheme presented here would suggest the alternative strategy of increasing the height of the energy gap through the enhancement of coherence, making it unchallengeable by the local productions of energy (i.e., improving the “terrain” as old medical doctors used to say). 

In summary, we think that a new perspective opens to biological research. It accepts all the achievements of modern molecular biology but embeds them within a collective dynamics originating from the basic quantum features of nature and is able to create an overall dynamic order in the organism. 
